# Supplementary material for: Upstream open reading frame translation enhances immunogenic peptide presentation in mitotically arrested cancer cells
Source: Nat Commun. 2025 Aug 27;16:8008. doi: 10.1038/s41467-025-63405-2 (PMC12391379; doi:10.1038/s41467-025-63405-2)
Supplement: Supplementary file 1 — Supplementary Information [file 41467_2025_63405_MOESM1_ESM.pdf]

Upstream open reading frame translation enhances immunogenic peptide presentation in mitotically arrested cancer cells

Alexander Kowar[1,2#], Jonas P. Becker[3,4#], Rossella Del Pizzo[1,2], Zhiwei Tang[1,2], Julien Champagne[5], Kathrin Wellach[2,3,6], Kiana Samimi[2,3,6], Ariel Galindo-Albarrán[7], Pierre-René Körner[5], Jasmine Montenegro Navarro[5], Andrés Elía[1], Fiona Megan Tilghman[1], Hanan Sakeer[1], Marco Antonio Mendoza-Parra[7], Angelika B. Riemer[3,6*], Reuven Agami[5,*], Fabricio Loayza-Puch[1,*]

#Equal contribution, *Correspondence to: a.riemer@dkfz.de; r.agami@nki.nl; f.loayza-puch@dkfz-heidelberg.de

1 Translational Control and Metabolism, German Cancer Research Center (DKFZ), Heidelberg, Germany.

2 Faculty of Biosciences, University of Heidelberg, Heidelberg, Germany

3 Division of Immunotherapy and Immunoprevention, German Cancer Research Center (DKFZ), Heidelberg, Germany

4 Immunopeptidomics Unit, National Center for Tumor Diseases (NCT), NCT Heidelberg, a partnership between DKFZ and University Hospital, Heidelberg, Germany

5 Division of Oncogenomics, Oncode Institute, The Netherlands Cancer Institute, Amsterdam, the Netherlands

6 Molecular Vaccine Design, German Center for Infection Research (DZIF), partner site Heidelberg, Heidelberg, Germany

7 UMR 8030 Génomique Métabolique, Genoscope, Institut François Jacob, CEA, CNRS, University of Evry-val-d'Essonne, University Paris-Saclay, 91057 Évry, France

Supplementary Figures 1 to 9

Supplementary Figure 1

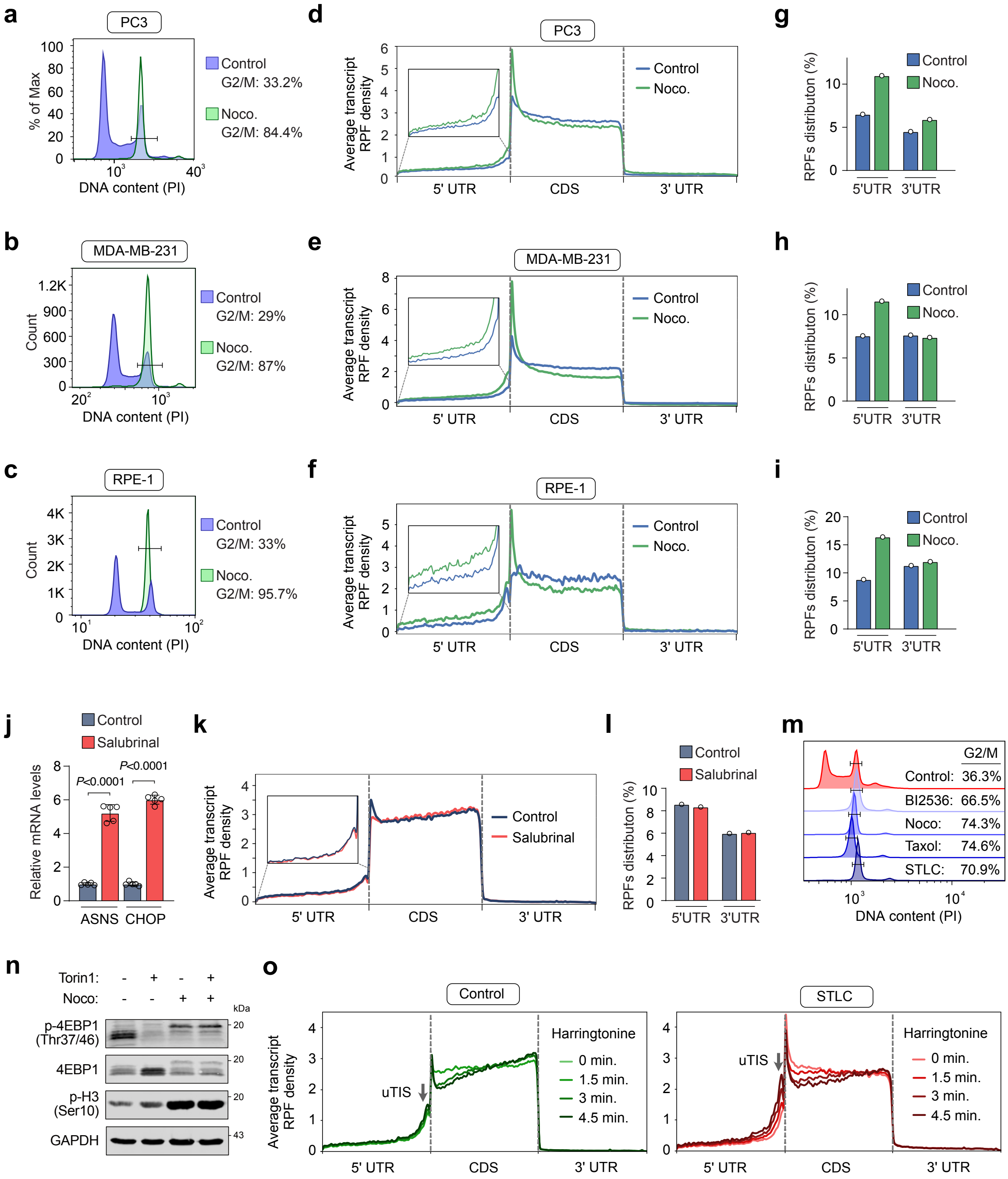

Supplementary Figure 1. Impact of mitotic arrest agents on RPF distribution in cancer cells.

a-c, Representative propidium iodide stainings of PC3 (a), MDA-MB-231 (b), and RPE-1 (c) cells arrested in mitosis with Nocodazole (Noco. 0.5 μM) for 16 hrs. Data from one experiment (*n*=1).

d-f, Metagene profiles of RPFs in proliferating and mitotically arrested PC3 (d), MDA-MB-231 (e), and RPE-1 (f) cells treated with Nocodazole (0.5 μM; 16 hrs). Data from one experiment (*n*=1).

g-i, Quantification of RPF distribution in the 5' UTR of PC3 (g), MDA-MB-231 (h), and RPE-1 (i) cells treated with vehicle (Control) or Nocodazole (0.5 μM) for 16 hrs.

j, qRT-PCR analysis of the indicated genes in U-2 OS cells treated with vehicle (Control) or Salubrinal (50 μM; 16 hrs.). Data represent mean ± SD from biologically independent experiments (*n*=5). *P*-values were calculated using a two-tailed unpaired *t*-test.

k, Metagene profiles of RPFs in U-2 OS cells treated with vehicle (Control) or Salubrinal (50 μM; 16 hrs.).

l, Quantification of RPF distribution in the 5' UTR and 3'UTR of U-2 OS cells treated with vehicle (Control) or Salubrinal (50 μM; 16 hrs.).

m, Representative propidium iodide stainings of U-2 OS cells treated with vehicle (Control) or BI2536. (0.1 μM), Nocodazole (0.5 μM), Taxol (1 μM), or STLC (5 μM) for 16 hrs.

n, Immunoblot of phospho-4E-BP1 (p-4E-BP1, Thr37/46) and total 4E-BP1 in U-2 OS cells treated with vehicle (Control), Nocodazole (0.5 μM), or Nocodazole (0.5 μM) + Torin1 (250 nM). Cells were treated with Nocodazole for 16 hrs. and with Torin1 for 2 hrs.

o, Metagene profiles of RPFs in MDA-MB-231 cells treated with vehicle (Control) or STLC (5 μM) for 16 hrs. Cells were harvested as described in Fig. 1i. uTIS, upstream translation initiation site.

Source data including exact *P*-values are provided as Source Data file.

Supplementary Figure 2

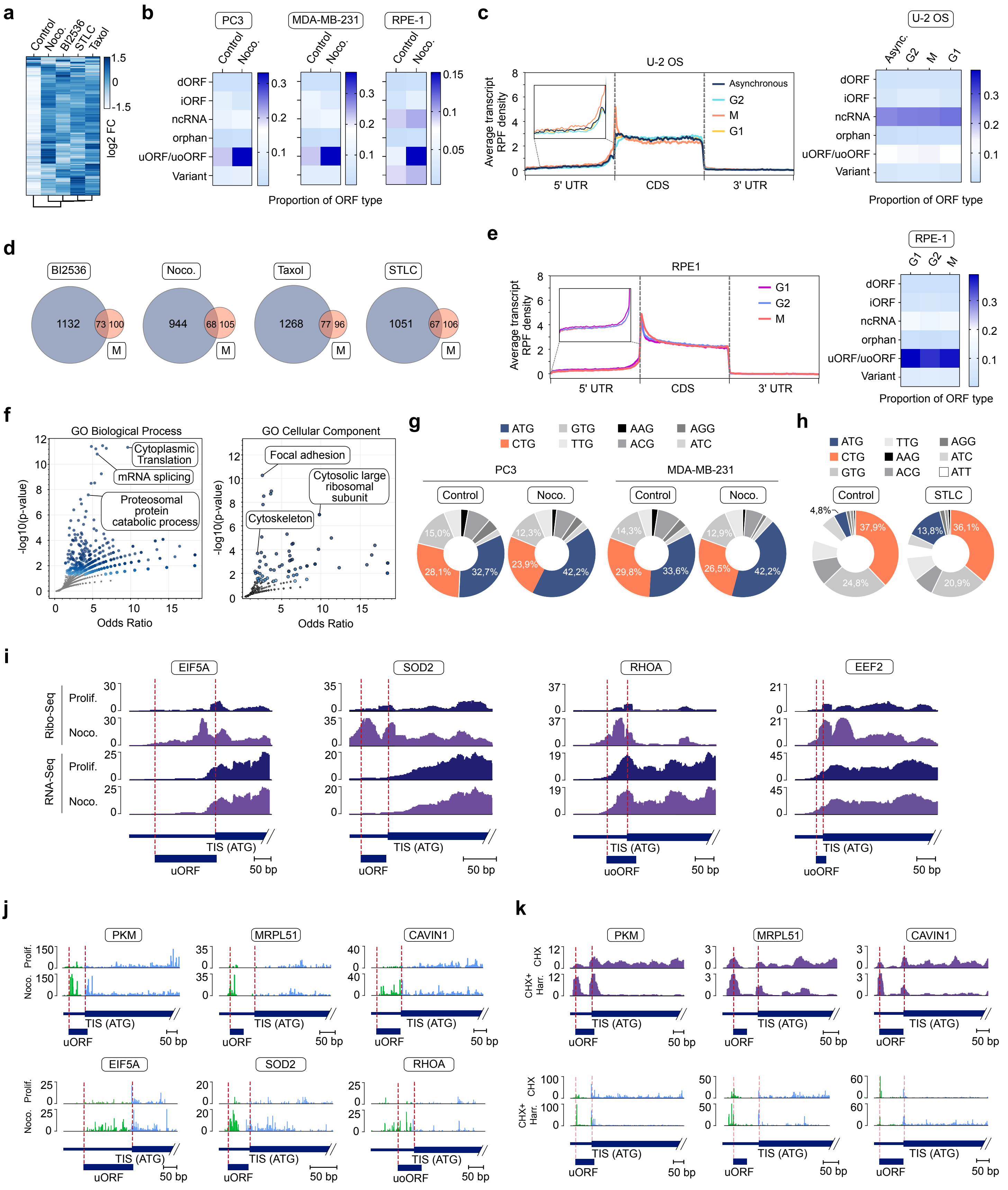

Supplementary Figure 2. Analysis of uORF/uoORF translation in mitotically arrested cancer cells

a, Heatmap showing normalized translation levels of predicted uORFs and uoORFs across all samples from Fig. 2b, based on PRICE-derived ORF annotations. Translation levels were normalized for library size using DESeq2, which was also used to identify ORFs significantly enriched in mitotic arrest compared to proliferating cells.

b, Proportion of ORF categories described in Fig. 2a in PC3 (left panel), MDA-MB-231 cells (middle panel), and RPE-1 cells (right panel) treated with vehicle (Control) or Nocodazole (0.5 µM) for 16 hrs.

c,e, Metagene profiles of ribosome-protected fragments (RPFs) in U-2 OS (panel c, left) and RPE-1 (panel d, left) cells synchronized in G1, M, and G2 phases. The right panels (c, e) display the distribution of ORF categories, as defined in Fig. 2a, for U-2 OS (c) and RPE-1 (e) under the same synchronization conditions. The inset focuses on the 5′ UTR to highlight region-specific changes in ribosome occupancy.

d, Venn diagram showing the number of uORFs/uoORFs identified by PRICE in U-2 OS cells arrested in mitosis with BI2536 (0.1 µM), Nocodazole (0.5 µM), Taxol (1 µM), or STLC (5 µM) for 16 hrs and U-2 OS cells synchronized in the mitotic phase (M).

f, Volcano plots showing enriched terms from the Gene Ontology (GO) Biological Process (left panel) and Cellular Component (right panel) for the common genes described in Fig. 2c. Each point represents a term, with the x-axis showing the odds ratio and the y-axis showing the log10(p-value). Larger and darker points signify terms with higher enrichment significance in the input gene set.

g, Percentage of uORF/uoORF translation initiation sites predicted by PRICE in PC3 (left panel) and MDA-MB-231 (right panel) cells treated with vehicle (Control) or Nocodazole (0.5 µM) for 16 hrs. The increase in uORFs/uoORFs with ATG initiation sites is 29% and 25.5% in mitotically arrested PC3 and MDA-MB-231 cells, respectively.

h, Percentage of uORF/uoORF translation initiation sites in U-2 OS cells treated with vehicle (Control) or STLC (5 µM) for 16 hrs. Counts were generated from harringtonine peaks identified in Fig. 1j

i, Representative examples of uORFs/uoORFs exhibiting elevated translational efficiency in Nocodazole-arrested U-2 OS cells. The upper panels show Ribo-Seq reads, while the lower panels show RNA-Seq reads for the 5′UTR and the initial region of the CDS. Prolif., proliferating; Noco., Nocodazole.

j, Representative P-site density plots from Ribo-seq data showing gene-level coverage, with uORFs highlighted in green.

k, Read distribution (upper panels) and P-site density plots (lower panels) from translation initiation site sequencing of representative uORFs/uoORFs in U-2 OS cells arrested in mitosis (STLC, 5 µM, 16 hrs.). CHX, Cycloheximide; Harr., Harringtonine.

Supplementary Figure 3

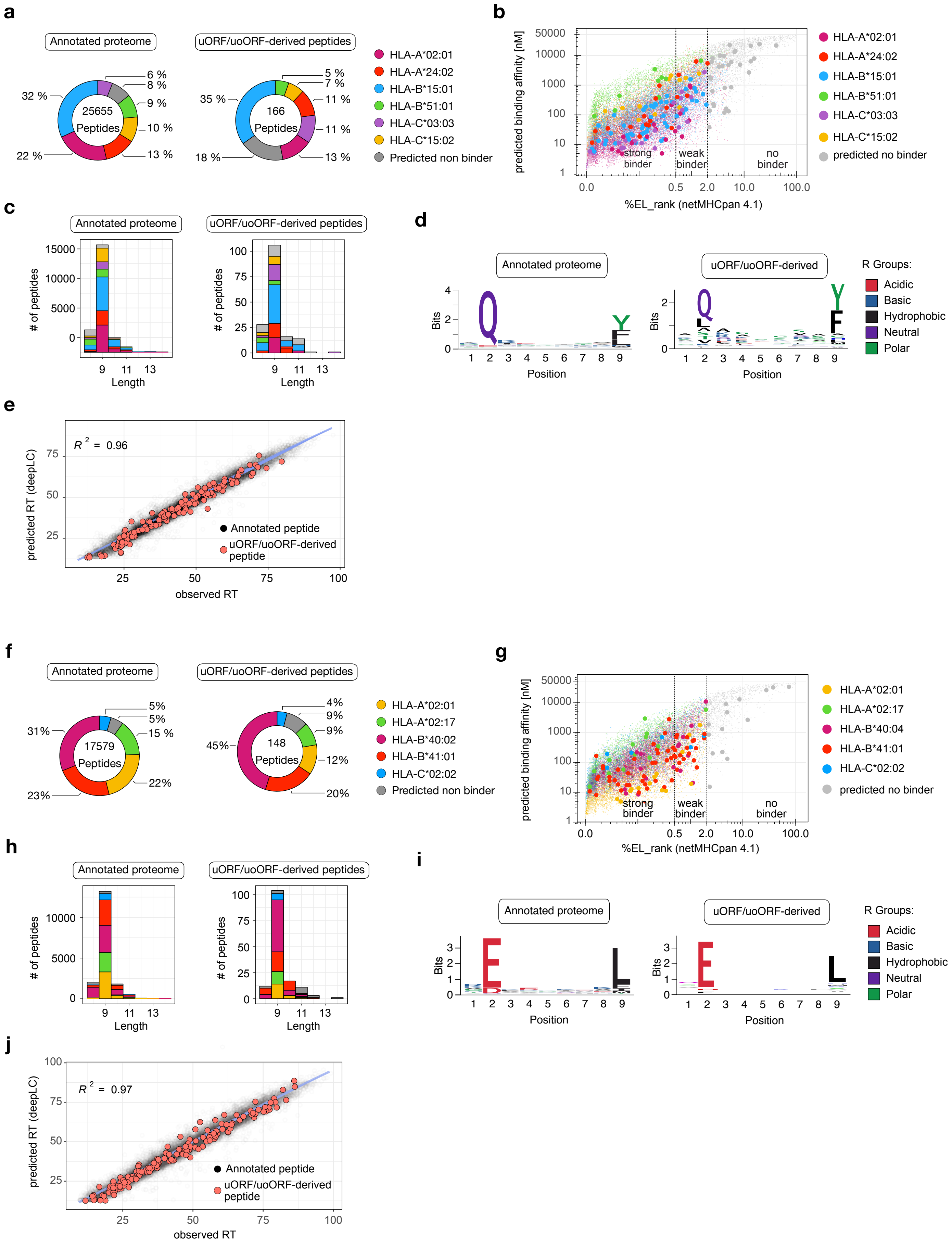

Supplementary Figure 3. Biochemical characteristics of annotated and uORF/uoORF-derived HLA-presented peptides in SUM-159PT and MDA-MB-231 cells.

a,f, Quantification of peptides in the annotated proteome (left panel) and uORF/uoORF-derived proteome (right panel) in proliferating and mitotically arrested SUM-159PT (a) and MDA-MB-231 (f) cells. The distribution of predicted binding to HLA alleles in both cell lines is presented.

b,g, Percentage of eluted ligand (EL) peptides predicted by NetMHCpan-4.1 plotted against predicted binding affinity, for peptides from the annotated proteome (small dots) and uORF/uoORF-derived peptides (large dots) of proliferating and mitotically arrested SUM-159PT (b) and MDA-MB-231 (g) cells. Predicted binding to HLA alleles in SUM-159PT (b) and MDA-MB-231 (g) cells is displayed. Peptides are classified as strong binders (%EL rank 0–0.5), weak binders (%EL rank 0.5–2), or non-binders (%EL rank 2–100).

c,h, Length distribution of detected peptides from the annotated proteome (left panel) and uORF/uoORF-derived peptides (right panel) in proliferating and mitotically arrested SUM-159PT (c) and MDA-MB-231 (h) cells. The proportion of predicted binding to HLA alleles in both cell lines is shown.

d,i, Peptide motif plots for unique peptides from the annotated proteome (4,402) and unique peptides derived from uORFs/uoORFs (59), confidently identified as binding to the SUM-159PT allele HLA-B*15:01 (d). Peptide motif plots for unique peptides from the annotated proteome (5,465) and unique peptides derived from uORFs/uoORFs (30), confidently identified as binding to the MDA-MB-231 allele HLA-B*40:02 (i)

e,j, Observed retention time (RT) indices plotted against predicted RT indices for peptides from the annotated (black) and uORF/uoORF-derived (red) proteomes, across all HLA alleles in proliferating and mitotically arrested SUM-159PT (e) and MDA-MB-231 (j) cells. R^2 indicates the Pearson correlation coefficient.

Supplementary Figure 4

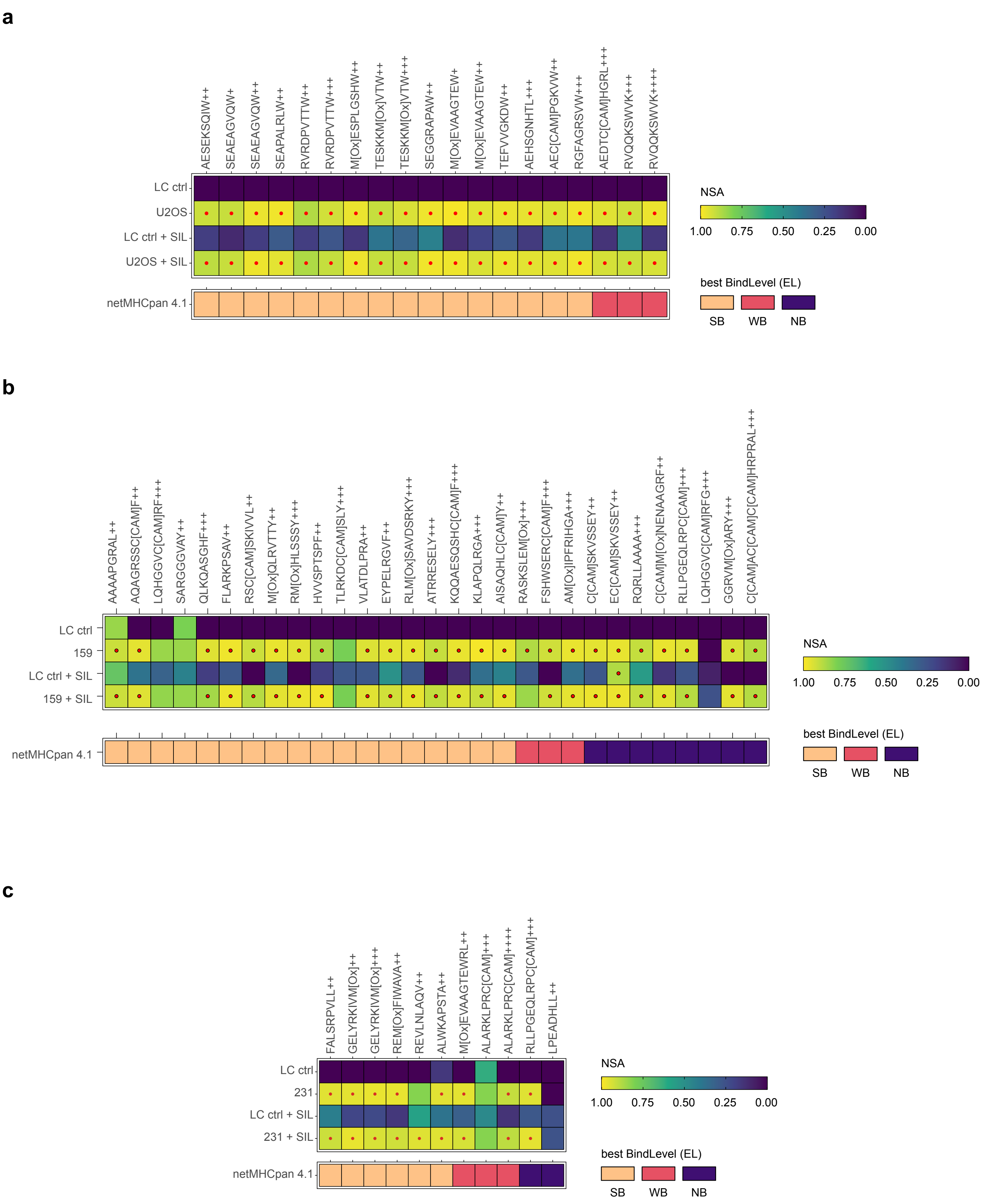

d

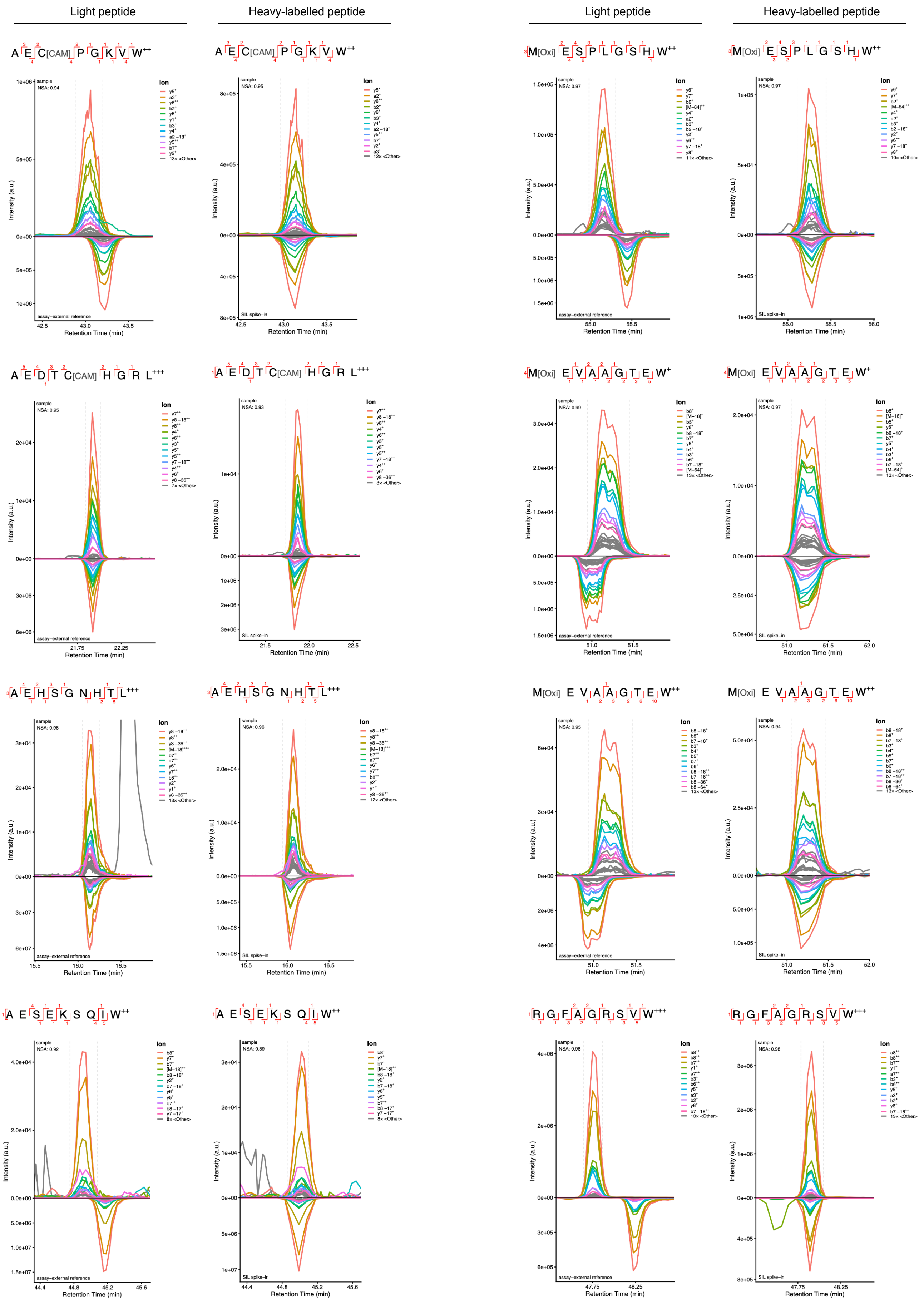
Light peptide
Heavy-labelled peptide
Light peptide
Heavy-labelled peptide
Intensity (a.u.)
Retention Time (min)
Ion
sample
assay–external reference
SIL spike–in

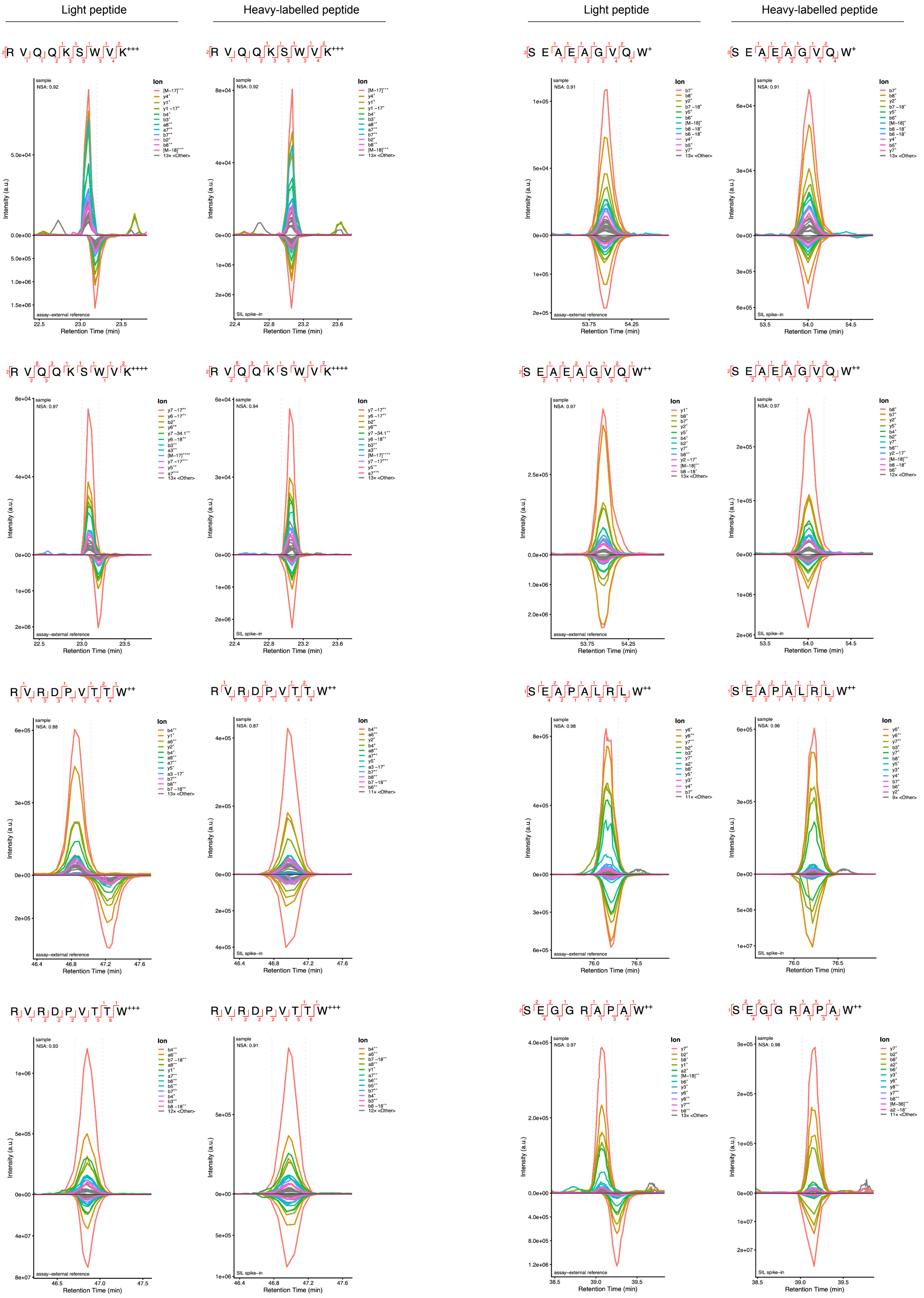
Light peptide
Heavy-labelled peptide
Light peptide
Heavy-labelled peptide
R V Q Q K S W V K+++
R V Q Q K S W V K++++
R V R D P V T T W++
R V R D P V T T W+++
S E A E A G V Q W+
S E A E A G V Q W++
S E A P A L R L W++
S E G G R A P A W++
sample
NSA: 0.92
NSA: 0.97
NSA: 0.94
NSA: 0.88
NSA: 0.87
NSA: 0.93
NSA: 0.91
NSA: 0.98
NSA: 0.96
Ion
Intensity (a.u.)
Retention Time (min)
assay–external reference
SIL spike–in

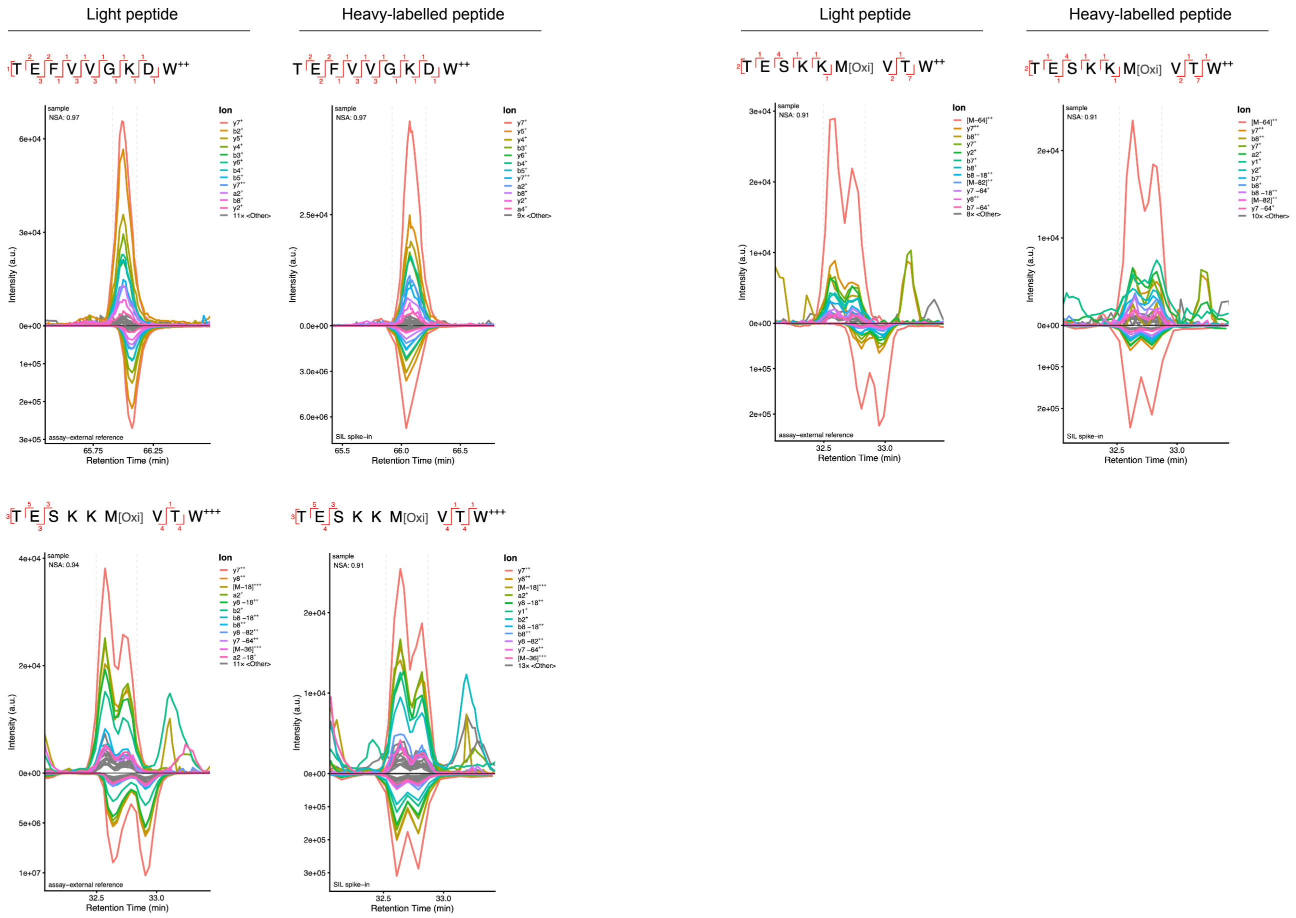
Light peptide
Heavy-labelled peptide
Light peptide
Heavy-labelled peptide
Ion
sample
assay–external reference
SIL spike–in
Intensity (a.u.)
Retention Time (min)

e

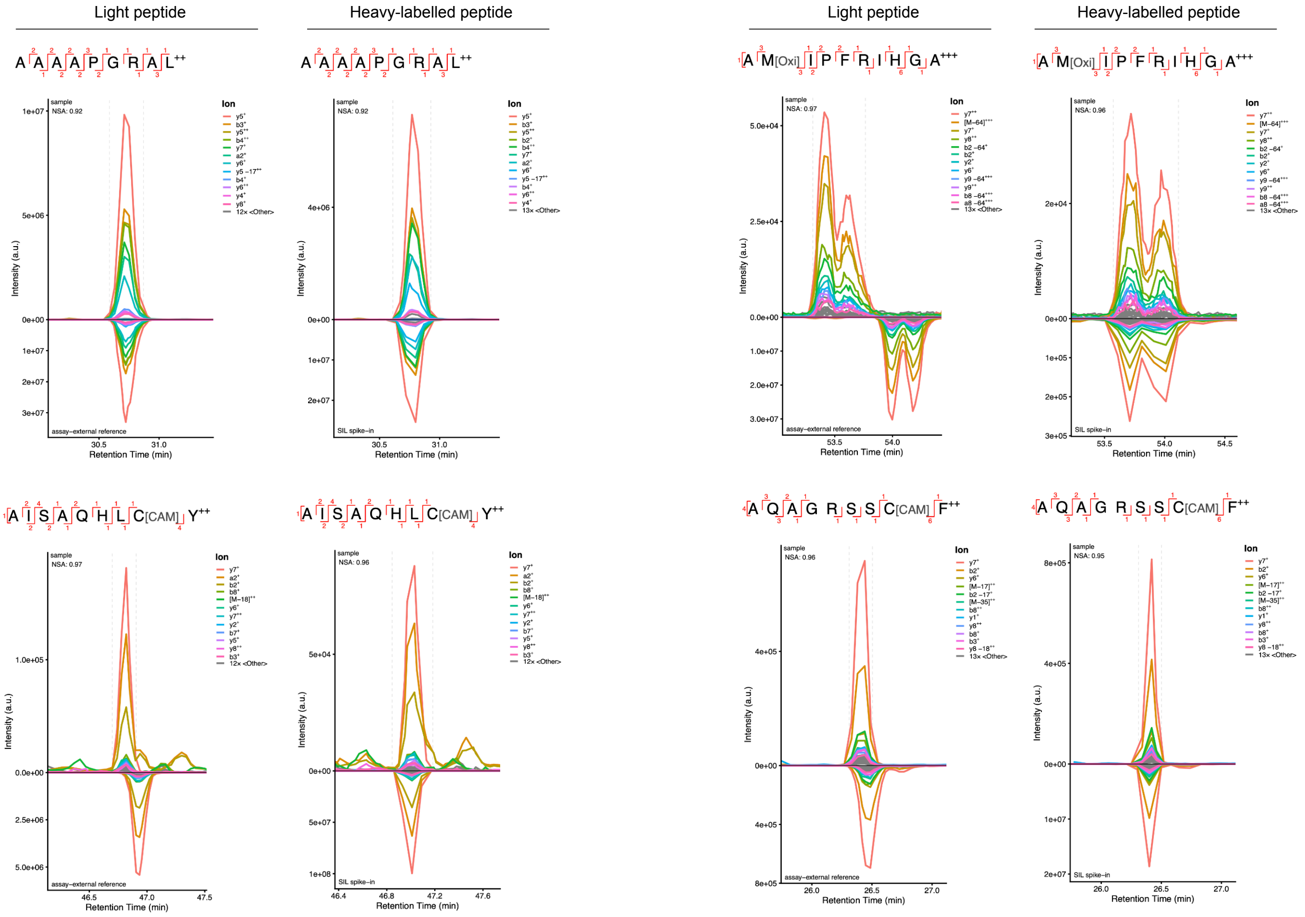
Light peptide
Heavy-labelled peptide
Light peptide
Heavy-labelled peptide
Ion
sample
assay–external reference
SIL spike–in
Intensity (a.u.)
Retention Time (min)

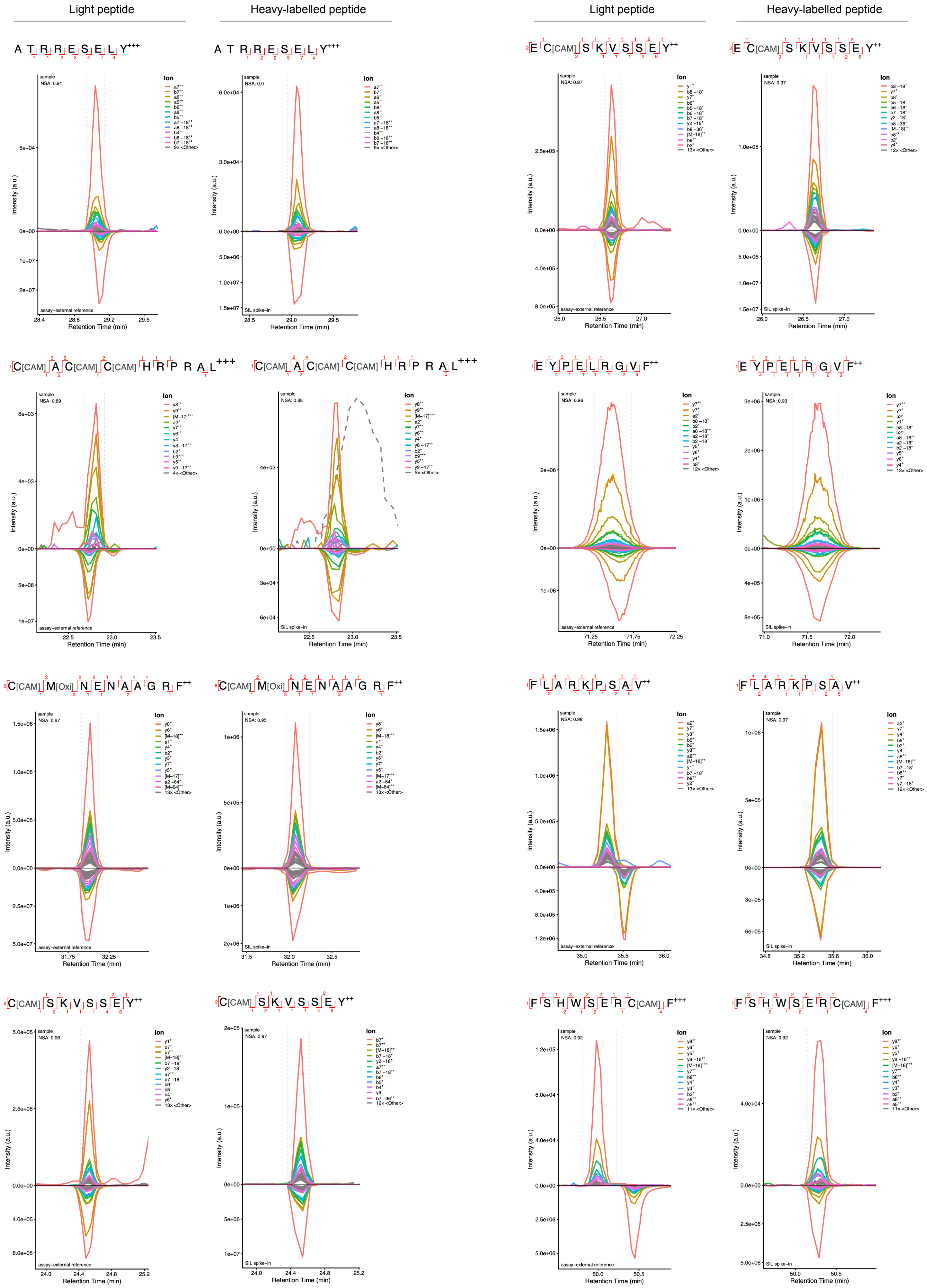
Light peptide
Heavy-labelled peptide
Light peptide
Heavy-labelled peptide
Intensity (a.u.)
Retention Time (min)
Ion
sample
assay–external reference
SIL spike–in

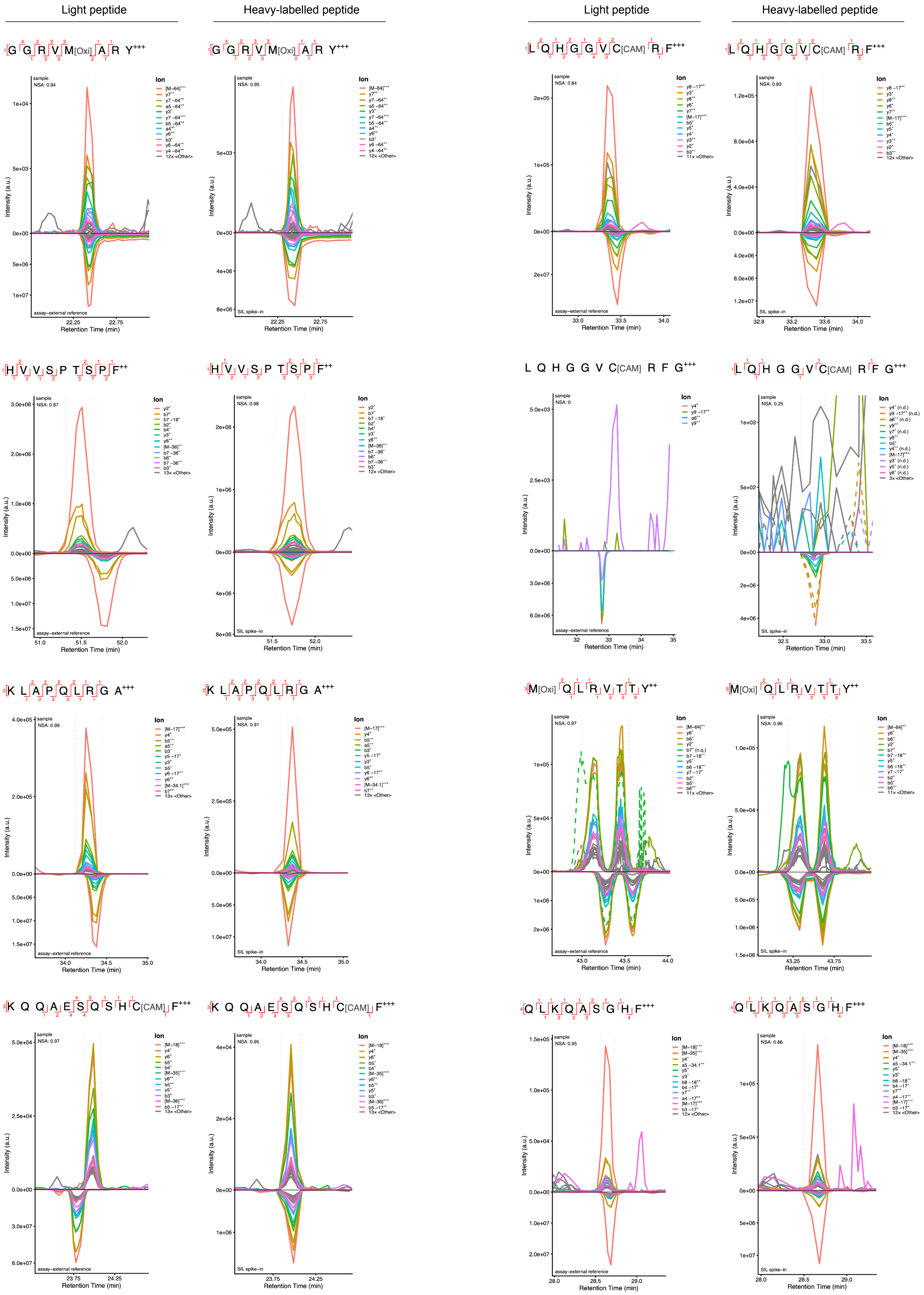

Light peptide
Heavy-labelled peptide
Light peptide
Heavy-labelled peptide
Intensity (a.u.)
Retention Time (min)
Ion
assay–external reference
SIL spike–in

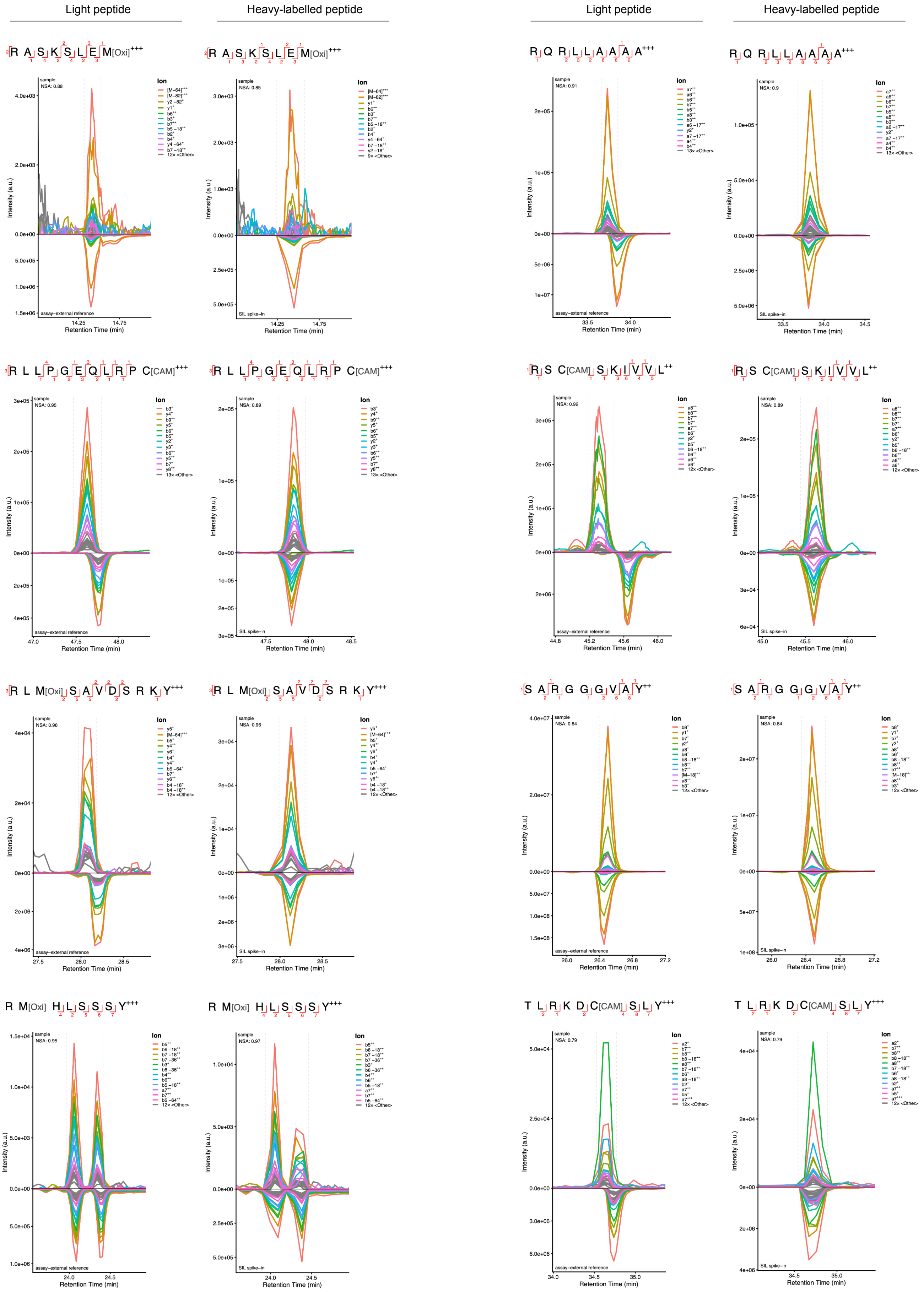
Light peptide
Heavy-labelled peptide
Light peptide
Heavy-labelled peptide
sample
NSA: 0.88
NSA: 0.85
NSA: 0.91
NSA: 0.9
NSA: 0.95
NSA: 0.89
NSA: 0.92
NSA: 0.89
NSA: 0.96
NSA: 0.96
NSA: 0.84
NSA: 0.84
NSA: 0.95
NSA: 0.97
NSA: 0.79
NSA: 0.79
Ion
Intensity (a.u.)
Retention Time (min)
assay–external reference
SIL spike–in

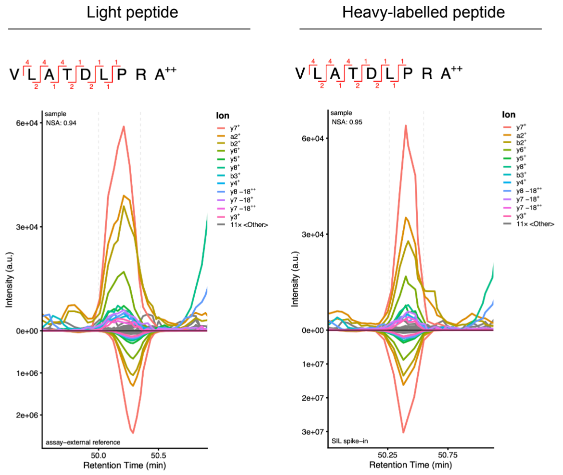

Light peptide
Heavy-labelled peptide
Intensity (a.u.)
Retention Time (min)
assay–external reference
SIL spike–in
Ion

f

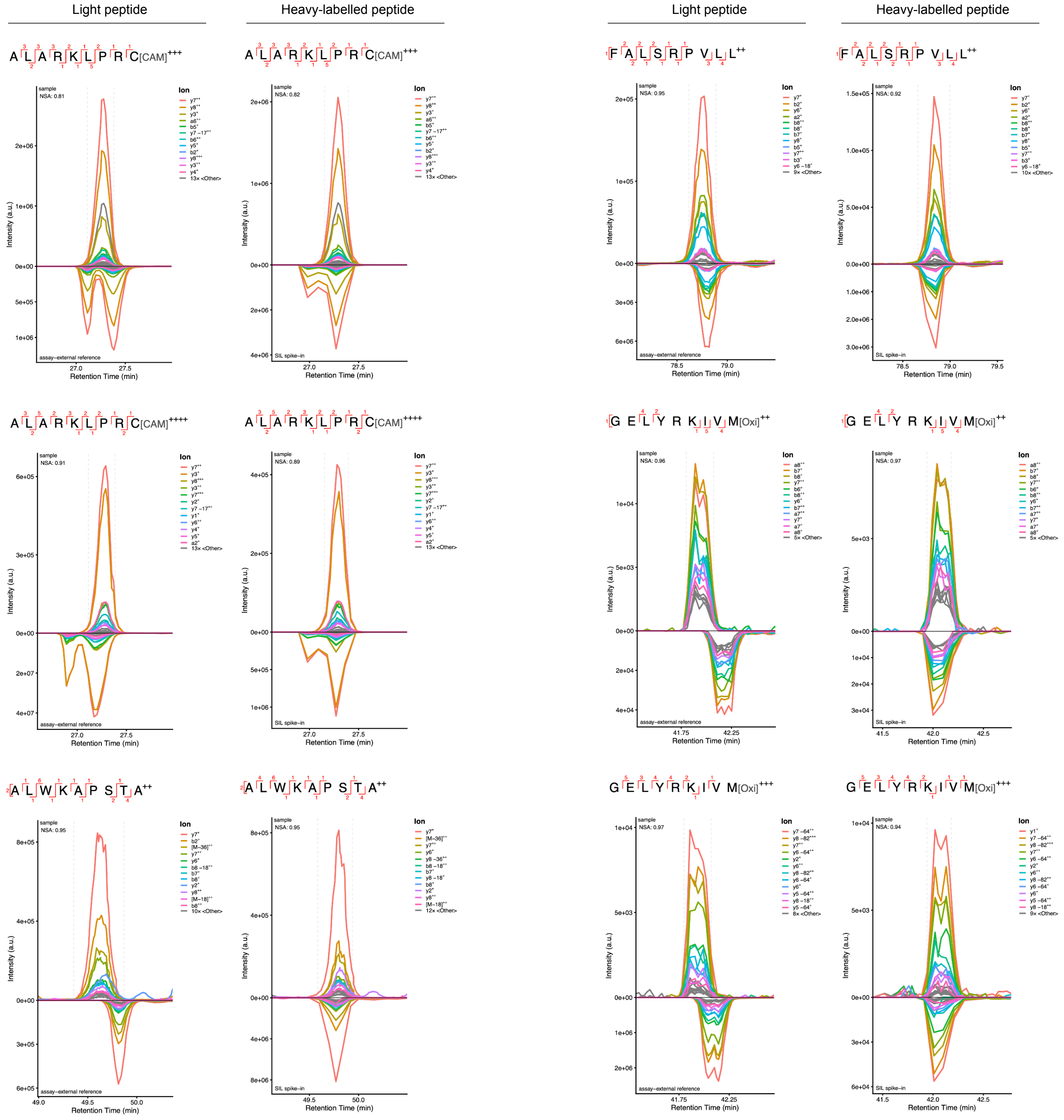

Light peptide
Heavy-labelled peptide
Light peptide
Heavy-labelled peptide
Intensity (a.u.)
Retention Time (min)
assay–external reference
SIL spike–in
Ion

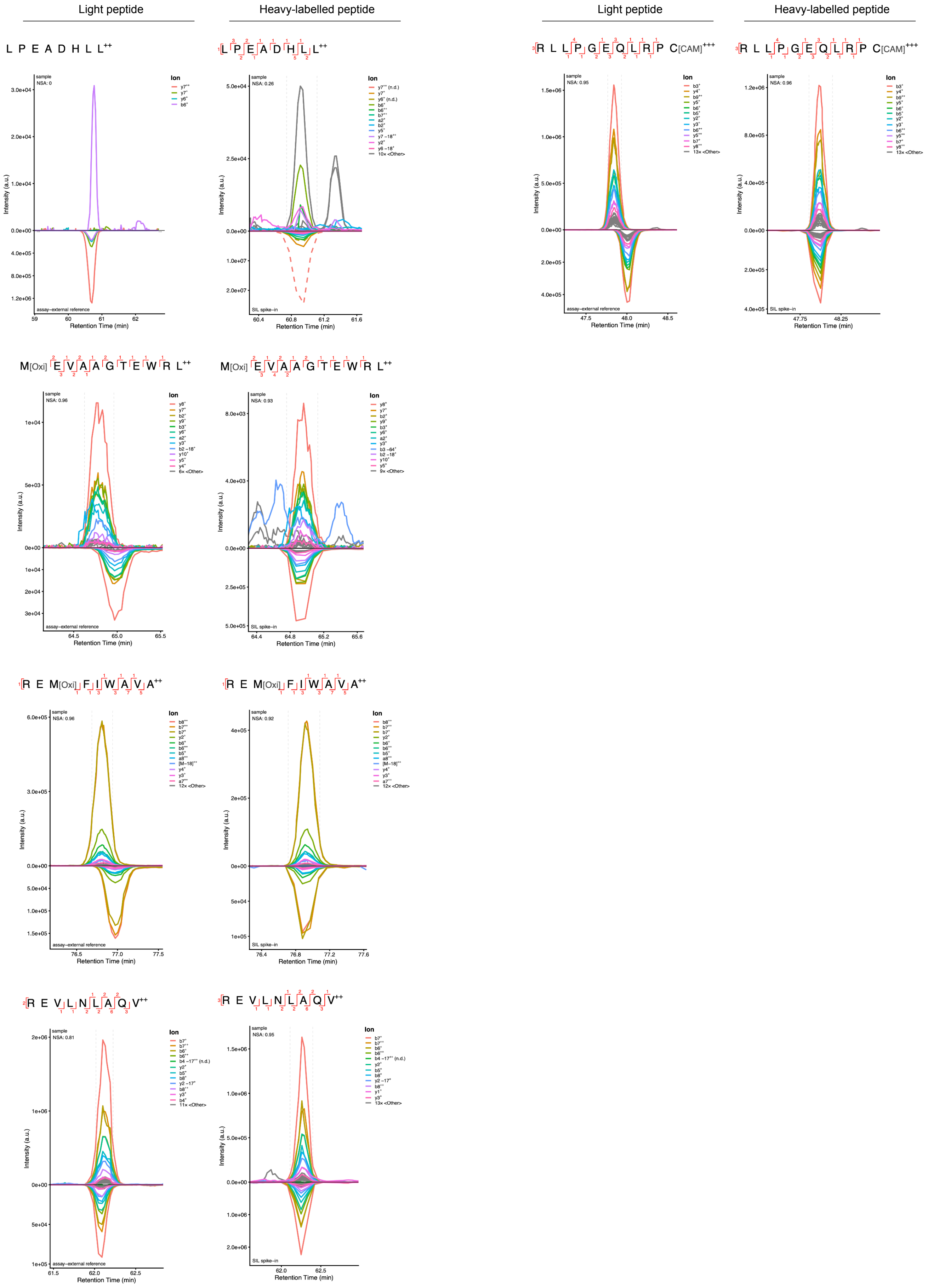

Light peptide
Heavy-labelled peptide
Light peptide
Heavy-labelled peptide
L P E A D H L L
R L L P G E Q L R P C[CAM]
M[Oxi] E V A A G T E W R L
R E M[Oxi] F I W A V A
R E V L N L A Q V
Ion
Intensity (a.u.)
Retention Time (min)
assay–external reference
SIL spike–in

Supplementary Figure 4. PRM validation of selected uORF/uoORF-derived peptides identified in cancer cells.

a-c, Heatmap overview of targeted LC-MS (PRM) analysis. Top candidate peptide sequences are indicated for U-2 OS (a), SUM-159PT (b), and MDA-MB-231 (c) cells. The normalized spectral contrast angle (NSA) was used to compare acquired fragment ion peaks to the reference library, with high-confidence detections (NSA ≥ 0.85) marked by a red dot.

d-f, Extracted ion chromatograms from optiPRM measurements without SIL spike-in comparing obtained signal to an assay-external reference (Light peptide) and with SIL spike-in comparing signal to the assay-internal reference (Heavy-labelled peptide) for peptides identified in U-2 OS (d), SUM-159PT (e), and MDA-MB-231 (f) cells.

Supplementary Figure 5

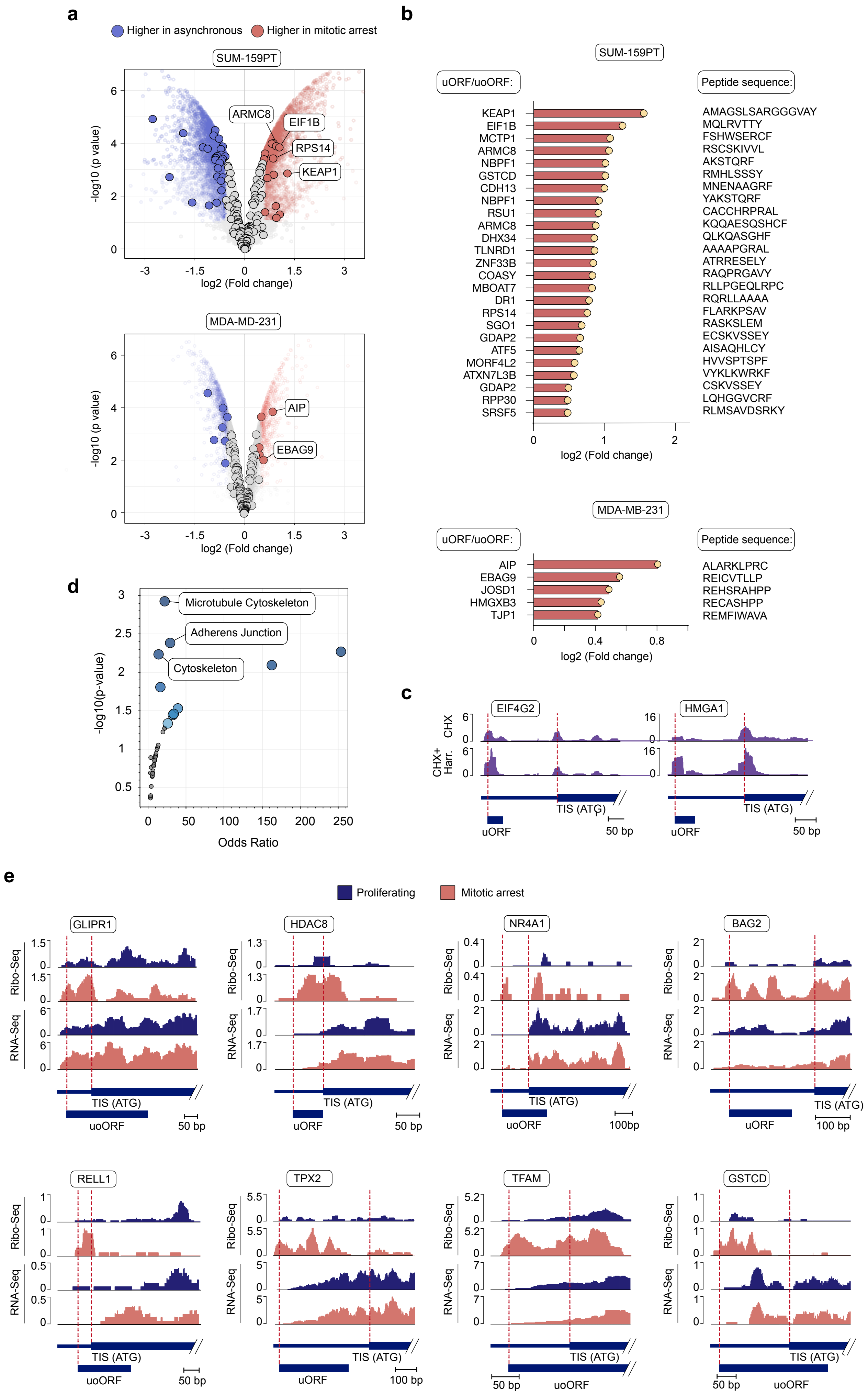

Supplementary Figure 5. Label-free quantification of HLA-presented uORF/uoORF-derived peptides in mitotically arrested cancer cells.

a, Volcano plot showing label-free quantification of the immunopeptidome in Taxol-treated versus DMSO-treated SUM-159PT (upper panel) and MDA-MB-231 cells (lower panel), highlighting the genes expressing uORF/uoORF-derived peptides. Peptides with a log2 fold change greater than 0.5 and adjusted *p*-value less than 0.05 are in red, while those with a log2 fold change below -0.5 and adjusted *p*-value less than 0.05 are in blue. The analysis was conducted using an empirical Bayes moderated *t*-test with two-sided *p*-values.

b, Mitotic arrest-induced uORF/uoORF-derived peptides in SUM-159PT (upper panel) and MDA-MB-231 (lower panel) cells, listing host gene name, log2 fold change (FC) of peptide abundance, and peptide sequences.

c, RPF reads distribution from translation initiation site sequencing of representative uORFs/uoORFs in U-2 OS cells arrested in mitosis. CHX, Cycloheximide. Harr, Harringtonine.

d, Gene Ontology (GO) Biological Process terms for the upregulated peptides in Fig. 4a, with each point representing a specific term. The x-axis displays the odds ratio, while the y-axis shows the -log10(*p*-value). Larger, darker points indicate terms with greater enrichment significance in the input gene set.

e, Read distribution profiles for uORFs/uoORFs exhibiting elevated expression in mitotically arrested U-2 OS cells. Ribo-Seq data (top tracks) and RNA-Seq data (bottom tracks) are shown, covering the 5' UTR and the initiation region of the coding sequence (CDS).

Supplementary Figure 6

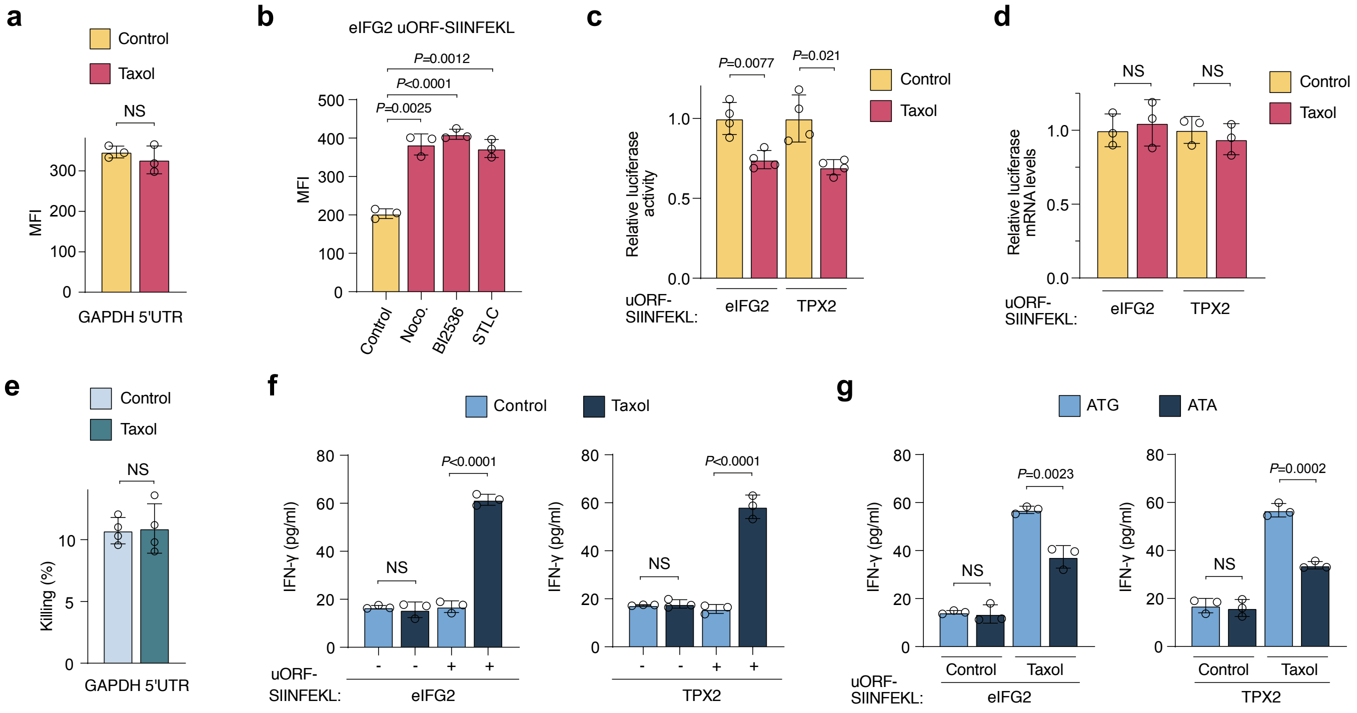

Supplementary Figure 6. Effect of mitotic arrest on peptide presentation and IFNγ production in TC1 cells.

a-b, Detection of the p:MHC complex SIINFEKL:H-2K^b by flow cytometry, shown as Median fluorescence intensity (MFI), in TC1 cells transfected with the indicated reporters. Cells were treated with vehicle (Control) or BI2536 (0.1 µM), Nocodazole (0.5 µM), or STLC (5 µM) for 16 hrs. Data represent mean ± SD from biologically independent experiments (n=3). P-values were calculated using a two-tailed unpaired t-test. NS, non-significant.

c-d, Activity assay (c) and qRT-PCR (d) of firefly luciferase in TC1 cells transfected with the indicated reporters. Cells were treated with vehicle (Control) or Taxol (1 µM) for 16 hrs. Data represent mean ± SD from biologically independent experiments (n=4 for c and n=3 for d). P-values were calculated using a two-tailed unpaired t-test. NS, non-significant.

e, *In vitro* cytotoxicity of activated OT-I cells against TC1 cells transfected with the indicated reporter. TC1 cells were either arrested in mitosis with 1 µM Taxol for 16 hrs. or treated with DMSO for the same duration. Bars represent mean ± SD from five biologically independent experiments (n=4). Statistical significance was determined by a two-tailed unpaired t-test; NS, not significant.

f-g, Quantification of IFN-γ levels produced by CD8+ OT-I T cells when co-cultured with TC1 cells. TC1 cells were transfected with the indicated uORF-SIINFEKL reporters and arrested in mitosis with Taxol (1 µM) for 16 hrs. Control cells were treated with DMSO for the same period. Data represent mean ± SD from biologically independent experiments (n=3). P-values were calculated using a two-tailed unpaired t-test. NS, non-significant.

Source data including exact P-values are provided as Source Data file.

Supplementary Figure 7

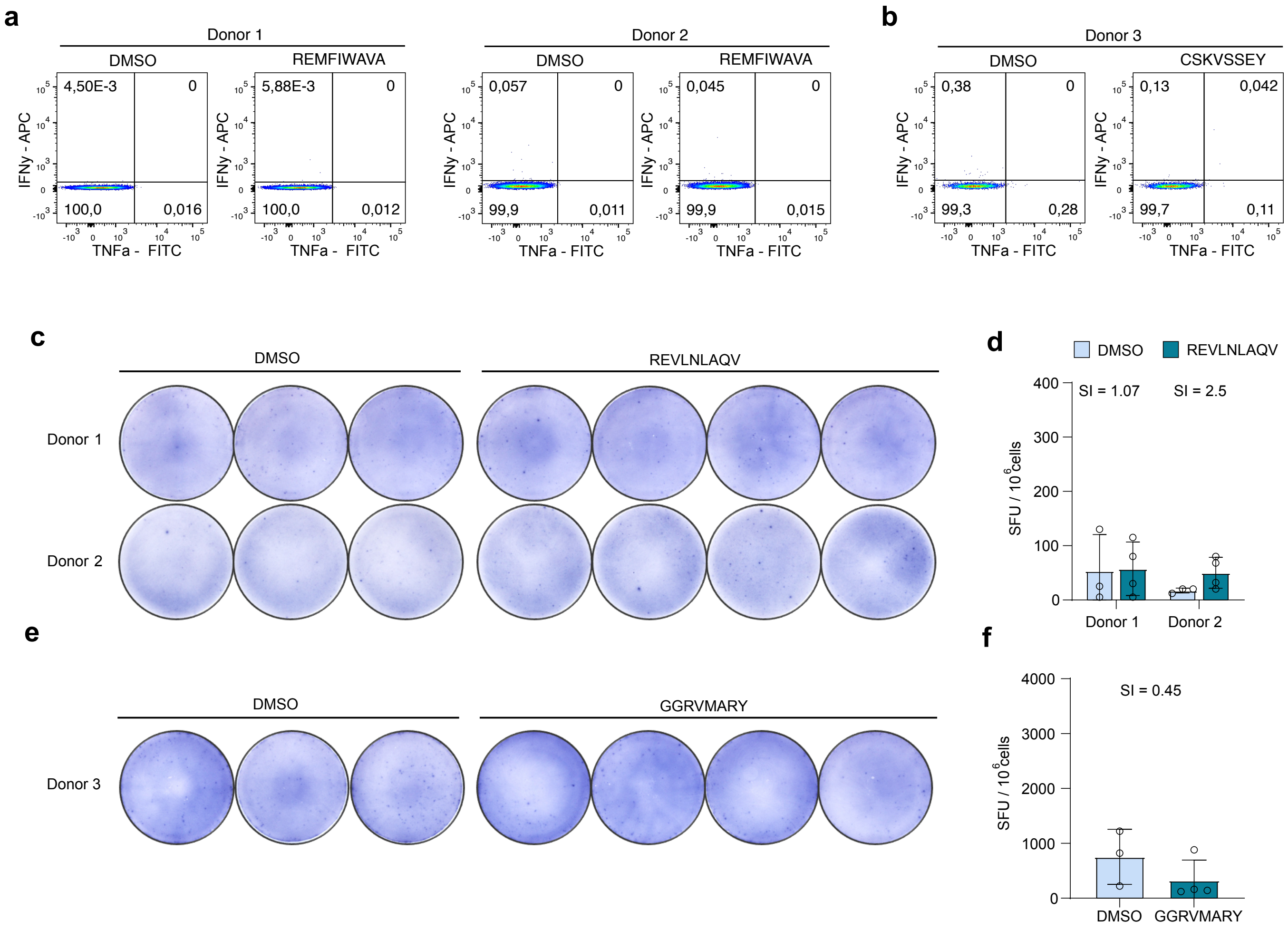

Supplementary Figure 7. CD4$^+$ T-cell responses to a uORF-derived peptide in healthy donors.

a-b, Expression of IFN-γ and TNF-α in CD4$^+$ T cells from healthy donors after treatment with DMSO or the uORF-derived peptides REMFIWAVA (a) and CSKVSSEY (b).

c,e, Representative ELISpot images showing IFN-γ secretion by PBMCs from three healthy donors following stimulation with the indicated peptides. DMSO served as the negative control.

d,f, ELISpot quantification corresponding to panels c and e, shown as spot-forming units (SFU) per 10^6 PBMCs. Data represent mean ± SD from technical replicates (*n*=3 for DMSO, *n*=4 for each peptide). SI, stimulation index.

Supplementary Figure 8

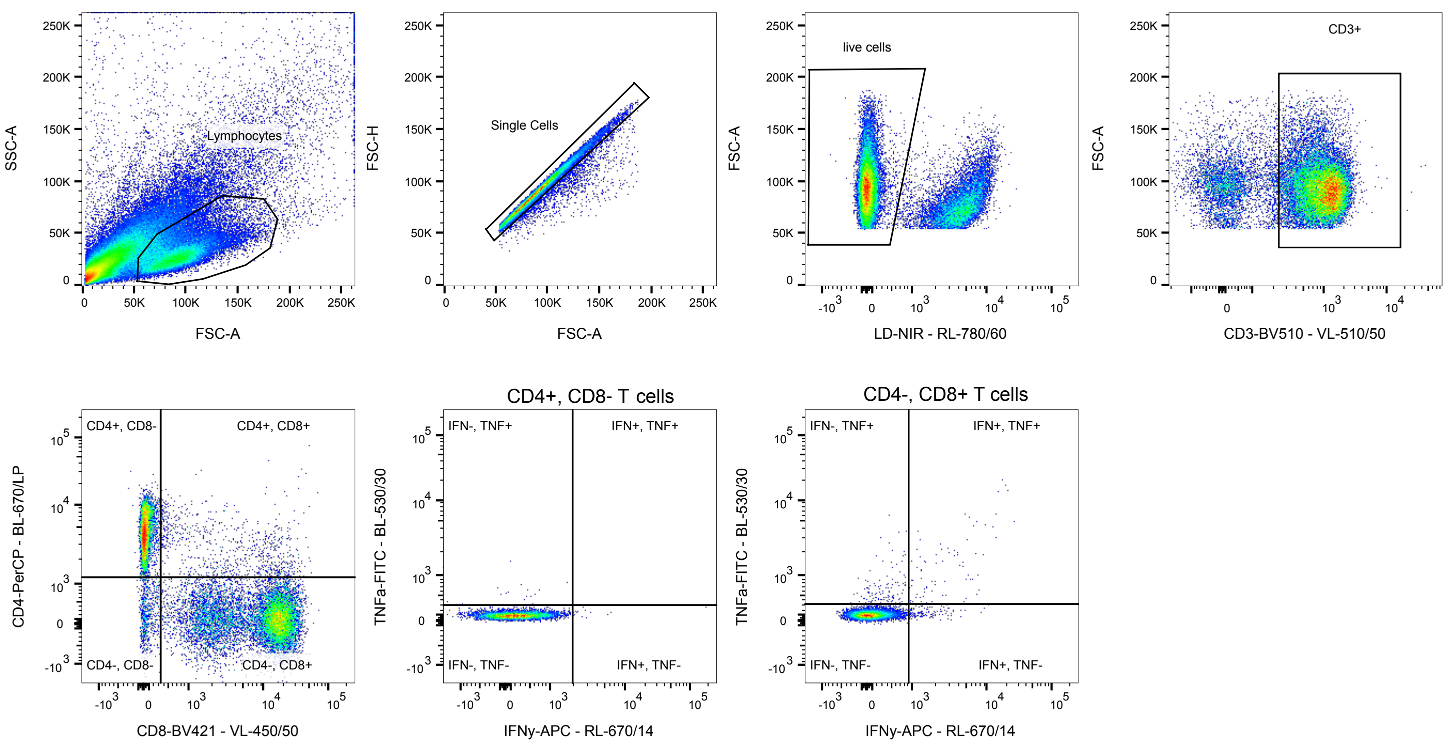

Supplementary Figure 8. Gating strategy for flow cytometry analysis.

Gating strategy used for Fig. 6 and Supplementary Fig. 7. Lymphocytes were gated based on forward scatter (FSC-A) versus side scatter (SSC-A). Singlets were then identified by gating FSC-A versus FSC-H. Viable cells were selected by excluding BL84/42-positive events (488 nm excitation). $CD3^+$ T cells were gated from viable singlets and subsequently subdivided into $CD4^+$ and $CD8^+$ subsets. Intracellular TNFα and IFNγ expression was assessed within both $CD4^+$ and $CD8^+$ populations using fluorescently labeled anti–TNFα and anti–IFNγ antibodies.

Supplementary Figure 9

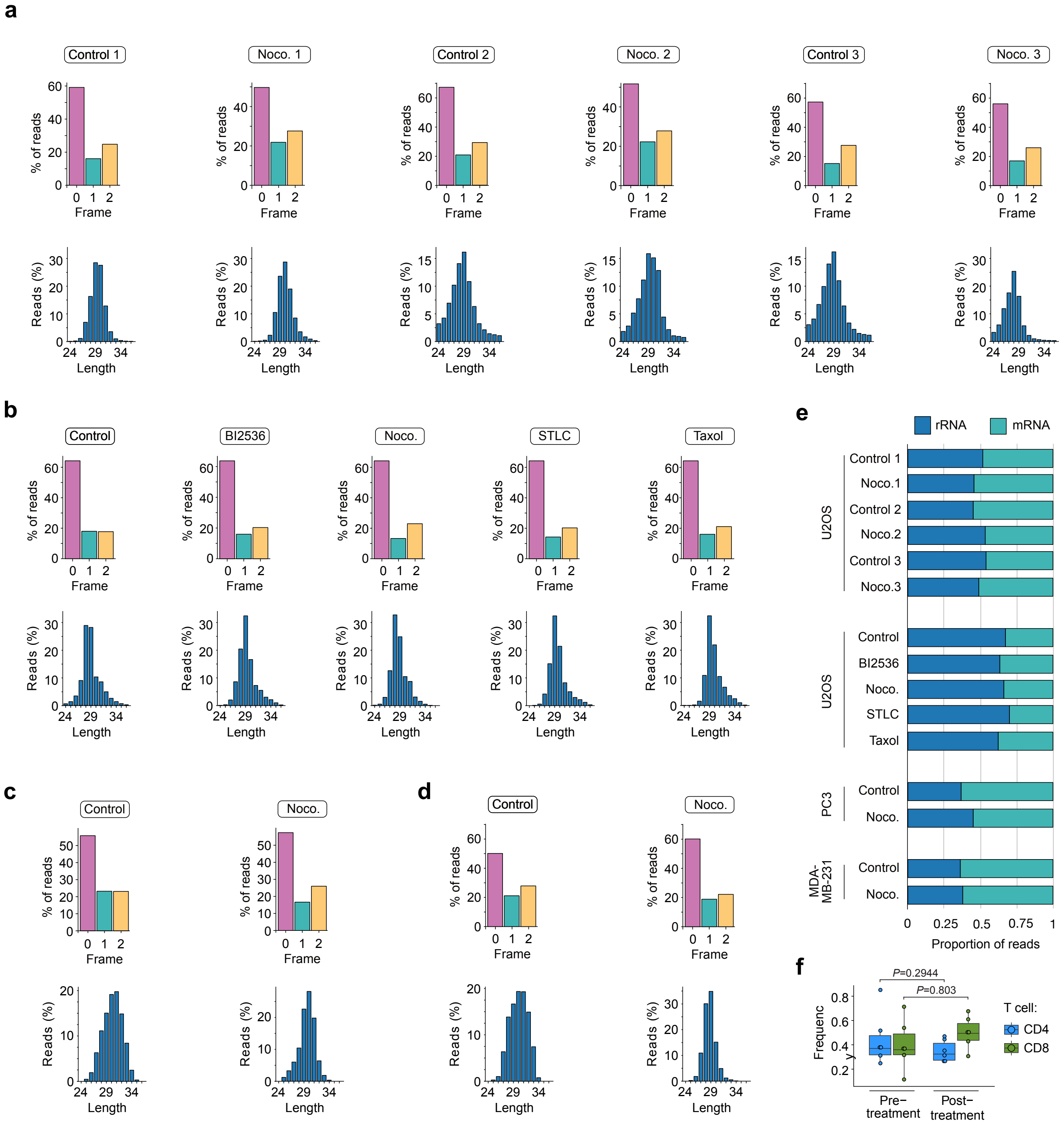

Supplementary Figure 9. Quality control analysis of ribosome profiling libraries.

a–d, In-frame read percentages (top panels) and ribosome-protected fragment (RPF) length distributions (bottom panels) for the libraries shown in Figure 1b–c (a), Figure 1e (b), Supplementary Figure 2d (c), and Supplementary Figure 2e (d).

e, Proportion of reads mapping to rRNA and mRNA in the Ribo-Seq libraries.

f, Boxplots showing the distribution of tumor-infiltrating $CD4^+$ and $CD8^+$ T-cell frequencies in scRNA-seq data from Paclitaxel-treated patients. Frequencies were calculated as the ratio of each T-cell subset to the total T-cell count per sample. Boxes indicate the median and interquartile range (IQR); whiskers extend to 1.5 × IQR. Statistical significance between groups was determined by two-sided Wilcoxon rank-sum tests.
