# Supplementary material for: Upstream open reading frame translation enhances immunogenic peptide presentation in mitotically arrested cancer cells
Source: Nat Commun. 2025 Aug 27;16:8008. doi: 10.1038/s41467-025-63405-2 (PMC12391379; doi:10.1038/s41467-025-63405-2)
Supplement: Supplementary file 2 — Description of Additional Supplementary Files [file 41467_2025_63405_MOESM2_ESM.pdf]

Description of Additional Supplementary Files

Supplementary Data 1. Actively translated non-canonical ORFs in U2OS cells treated with DMSO for 16 hours.

Supplementary Data 2. Actively translated non-canonical ORFs in U2OS cells treated with Nocodazole for 16 hours.

Supplementary Data 3. Actively translated non-canonical ORFs in U2OS cells treated with STLC for 16 hours.

Supplementary Data 4. Actively translated non-canonical ORFs in U2OS cells treated with Taxol for 16 hours.

Supplementary Data 5. Actively translated non-canonical ORFs in U2OS cells treated with BI2536 for 16 hours.

Supplementary Data 6. Actively translated non-canonical ORFs in PC3 cells treated with DMSO for 16 hours

Supplementary Data 7. Actively translated non-canonical ORFs in PC3 cells treated with Nocodazole for 16 hours.

Supplementary Data 8. Actively translated non-canonical ORFs in MDA-MB-231 cells treated with DMSO for 16 hours.

Supplementary Data 9. Actively translated non-canonical ORFs in MDA-MB-231 cells treated with Nocodazole for 16 hours.

Supplementary Data 10. Actively translated non-canonical ORFs in RPE-1 cells treated with DMSO for 16 hours

Supplementary Data 11. Actively translated non-canonical ORFs in RPE-1 cells treated with Nocodazole for 16 hours

Supplementary Data 12. Database of uORF/uoORFs sequences used as a reference for MS-immunopeptidomics data analysis

Supplementary Data 13. Oligonucleotides used in this study
