# Supplementary material for: Upstream open reading frame translation enhances immunogenic peptide presentation in mitotically arrested cancer cells
Source: Nat Commun. 2025 Aug 27;16:8008. doi: 10.1038/s41467-025-63405-2 (PMC12391379; doi:10.1038/s41467-025-63405-2)
Supplement: Supplementary file 16 — Reporting Summary [file 41467_2025_63405_MOESM16_ESM.pdf]

nature portfolio

Corresponding author(s): Fabricio Loayza-Puch

Last updated by author(s): Jun 10, 2025

Reporting Summary

Nature Portfolio wishes to improve the reproducibility of the work that we publish. This form provides structure for consistency and transparency in reporting. For further information on Nature Portfolio policies, see our Editorial Policies and the Editorial Policy Checklist.

Statistics

For all statistical analyses, confirm that the following items are present in the figure legend, table legend, main text, or Methods section.

n/a	Confirmed	
☐	☒	The exact sample size (*n*) for each experimental group/condition, given as a discrete number and unit of measurement
☒	☐	A statement on whether measurements were taken from distinct samples or whether the same sample was measured repeatedly
☐	☒	The statistical test(s) used AND whether they are one- or two-sided<br>*Only common tests should be described solely by name; describe more complex techniques in the Methods section.*
☐	☒	A description of all covariates tested
☒	☐	A description of any assumptions or corrections, such as tests of normality and adjustment for multiple comparisons
☐	☒	A full description of the statistical parameters including central tendency (e.g. means) or other basic estimates (e.g. regression coefficient) AND variation (e.g. standard deviation) or associated estimates of uncertainty (e.g. confidence intervals)
☐	☒	For null hypothesis testing, the test statistic (e.g. *F*, *t*, *r*) with confidence intervals, effect sizes, degrees of freedom and *P* value noted<br>*Give P values as exact values whenever suitable.*
☒	☐	For Bayesian analysis, information on the choice of priors and Markov chain Monte Carlo settings
☒	☐	For hierarchical and complex designs, identification of the appropriate level for tests and full reporting of outcomes
☐	☒	Estimates of effect sizes (e.g. Cohen's *d*, Pearson's *r*), indicating how they were calculated

Our web collection on statistics for biologists contains articles on many of the points above.

Software and code

Policy information about availability of computer code

Data collection	Flow cytometric analysis was performed on a FACSCanto Analyzer (BD Biosciences, New Jersey, USA).<br><br>Ribo-seq libraries were prepared as previously described (see Methods section) and sequenced single-end, 100 bp, on one lane of a Nextseq 2000 sequencers (Illumina).<br>qPCR data were acquired using QuantStudio 3 Real-Time PCR System.<br><br>Flow cytometry data were analyzed using FACSDiva Software Version 6, Cytek Spectroflaw and Flowlo 10.4 (BD Biosciences).<br><br>FAIMS-DIA MS raw data was analyzed using Spectronaut software (version 17.6; Biognosys)<br><br>uORF/uoORF-derived peptide sequences were manually validated using Skyline (version 22)
Data analysis	Differential gene expression analysis was performed with the R package DESeq2 v1.26.0

For manuscripts utilizing custom algorithms or software that are central to the research but not yet described in published literature, software must be made available to editors and reviewers. We strongly encourage code deposition in a community repository (e.g. GitHub). See the Nature Portfolio guidelines for submitting code & software for further information.

Data

Policy information about availability of data

All manuscripts must include a data availability statement. This statement should provide the following information, where applicable:

- Accession codes, unique identifiers, or web links for publicly available datasets
- A description of any restrictions on data availability
- For clinical datasets or third party data, please ensure that the statement adheres to our policy

The sequence data from this study have been submitted to the GEO repository: GSE281253 (https://www.ncbi.nlm.nih.gov/geo/query/acc.cgi?acc=GSE281253).

The mass spectrometry proteomics data have been deposited to the ProteomeXchange Consortium via the PRIDE partner repository with the dataset identifier PXD057839.

Research involving human participants, their data, or biological material

Policy information about studies with human participants or human data. See also policy information about sex, gender (identity/presentation), and sexual orientation and race, ethnicity and racism.

Reporting on sex and gender	Sex/gender was not considered in the experimental design.
Reporting on race, ethnicity, or other socially relevant groupings	N/A
Population characteristics	N/A
Recruitment	Experiments were performed with residual blood samples obtained from healthy platelet donors of the Hannover Medical School (MHH) Institute of Transfusion Medicine and Transplant Engineering of Hannover Medical School
Ethics oversight	Ethical vote number: 3639-2017

Note that full information on the approval of the study protocol must also be provided in the manuscript.

Field-specific reporting

Please select the one below that is the best fit for your research. If you are not sure, read the appropriate sections before making your selection.

☒ Life sciences ☐ Behavioural & social sciences ☐ Ecological, evolutionary & environmental sciences

For a reference copy of the document with all sections, see nature.com/documents/nr-reporting-summary-flat.pdf

Life sciences study design

All studies must disclose on these points even when the disclosure is negative.

Sample size	Sample sizes were determined based on prior knowledge of good sample sizes to ensure adequate data for reliable assessments as well as feasibility sequencing data generation. Sample sizes are indicated in the figure legends.
Data exclusions	Data was excluded based on QC results for individual assays.
Replication	Most experiments were performed in at least three biological replicates and specific sample sizes are mentioned in the figure legends. Most experiments contain statistical analysis and significances of the results are indicated.
Randomization	N/A
Blinding	The investigators were not blinded to group allocation due to fixed experimental groups used for comparison.

Reporting for specific materials, systems and methods

We require information from authors about some types of materials, experimental systems and methods used in many studies. Here, indicate whether each material, system or method listed is relevant to your study. If you are not sure if a list item applies to your research, read the appropriate section before selecting a response.

Materials & experimental systems

n/a	Involved in the study
	[x] Antibodies
	[x] Eukaryotic cell lines
[x]	Palaeontology and archaeology
	[x] Animals and other organisms
[x]	Clinical data
[x]	Dual use research of concern
[x]	Plants

Methods

n/a	Involved in the study
[x]	ChIP-seq
	[x] Flow cytometry
[x]	MRI-based neuroimaging

Antibodies

Antibodies used	4E-BP1 (Cell Signaling; cat. #9644, 1:1,000), Phospho-4E-BP1 (Thr37/46) (Cell Signaling; cat. #2855, 1:1,000), Phospho-Histone H3 (Ser10) (Cell Signaling; cat. #9701, 1:1,000), GAPDH (ProteinTech; cat. #60004; 1:20,000).
Validation	Antibodies were purchased from the above stated companies. Antibodies are well described and published elsewhere. information can be sought from the manufactures website under the respective catalog number.

Eukaryotic cell lines

Policy information about cell lines and Sex and Gender in Research

Cell line source(s)	U2OS (ATCC, HTB-96), SUM-159PT (BioIVT, CVCL_5423), MDA-MD-231 (ATCC ,HTB-26), PC3 (ATCC, CRL-1435), TC1 (ATCC, CRL-2785), RPE-1 (ATCC, CRL4000)
Authentication	All cell lines were authenticated using the Multiplex cell line authentication test by Multiplexion (Heidelberg Germany)
Mycoplasma contamination	All cell lines were controlled for contamination using the LookOut Mycoplasma PCR Detection Kit (Sigma)
Commonly misidentified lines (See ICLAC register)	No commonly misidentified cell lines were used.

Animals and other research organisms

Policy information about studies involving animals; ARRIVE guidelines recommended for reporting animal research, and Sex and Gender in Research

Laboratory animals	Mus musculus, C57BL/6, 7-8 weeks old.
Wild animals	No wild animal we used in this study
Reporting on sex	N/A
Field-collected samples	No field-collected samples were used in this study
Ethics oversight	OT-1 female mice (Clarke, S. R. et al., 2000) (6–8 weeks old) were bred in-house at our animal facility. All animals were housed under standardized laboratory conditions. To reduce environmental variation, experimental and control mice were co-housed. The facility maintained a 12-hour light/dark cycle, with ambient temperature controlled at 22±2°C and relative humidity between 45% and 65%. Mice had ad libitum access to food and water and were housed in groups of 3 to 5 per cage. All animals were maintained in a specific pathogen-free (SPF) environment and handled in accordance with institutional and governmental regulations, as approved by the German Cancer Research Center and the regional authority (Regierungspräsidium Karlsruhe; G57-20), following the German Animal Protection Law and the European Directive 2010/63/EU on the protection of animals used for scientific purposes.

Note that full information on the approval of the study protocol must also be provided in the manuscript.

Plants

Seed stocks	*Report on the source of all seed stocks or other plant material used. If applicable, state the seed stock centre and catalogue number. If plant specimens were collected from the field, describe the collection location, date and sampling procedures.*
Novel plant genotypes	*Describe the methods by which all novel plant genotypes were produced. This includes those generated by transgenic approaches, gene editing, chemical/radiation-based mutagenesis and hybridization. For transgenic lines, describe the transformation method, the number of independent lines analyzed and the generation upon which experiments were performed. For gene-edited lines, describe the editor used, the endogenous sequence targeted for editing, the targeting guide RNA sequence (if applicable) and how the editor was applied.*
Authentication	*Describe any authentication procedures for each seed stock used or novel genotype generated. Describe any experiments used to assess the effect of a mutation and, where applicable, how potential secondary effects (e.g. second site T-DNA insertions, mosiacism, off-target gene editing) were examined.*

Flow Cytometry

Plots

Confirm that:

☒ The axis labels state the marker and fluorochrome used (e.g. CD4-FITC).

☒ The axis scales are clearly visible. Include numbers along axes only for bottom left plot of group (a 'group' is an analysis of identical markers).

☒ All plots are contour plots with outliers or pseudocolor plots.

☒ A numerical value for number of cells or percentage (with statistics) is provided.

Methodology

Sample preparation	Sample preparation of the individual flow cytometry experiments are described in detail in the methods part of the manuscript.
Instrument	FACS Canto II cytometer, Fortessa HTS, BD Aria II (BD Biosciences)
Software	FlowJo 10.0.8 (BD)
Cell population abundance	*Describe the abundance of the relevant cell populations within post-sort fractions, providing details on the purity of the samples and how it was determined.*
Gating strategy	Gating strategies are described in the manuscript

☒ Tick this box to confirm that a figure exemplifying the gating strategy is provided in the Supplementary Information.
